# A 2-year-old with a hepatic abscess secondary to an ascending retrocecal appendicitis: case report and review of the literature

**DOI:** 10.1186/s12245-019-0260-9

**Published:** 2019-12-19

**Authors:** Gregory M. Taylor, Ethan R. Saffer, Eric L. McDowell, Matthew A. Warpinski

**Affiliations:** 1 D.O. Assistant Professor of Clinical Emergency Medicine at Indiana University School of Medicine, On staff at Ball Memorial Hospital, Department of Emergency Medicine, 2401 W. University Ave, Muncie, IN USA; 2 D.O. Assistant Clinical Professor, Beaumont Hospital, Botsford Campus, Teaching hospital of Michigan State University, Department of Emergency Medicine, Farmington Hills, MI USA

**Keywords:** Sepsis, Pediatrics, Hepatic abscess, Retrocecal, Appendicitis

## Abstract

**Background:**

Diagnosing appendicitis within the pediatric population can be challenging, whether it be a neonate with irritability or a toddler with flank pain. Symptoms may mimic a viral illness, constipation, urinary tract infection, or intussusception, all of which are more common in this age group when compared with appendicitis. While a ruptured appendicitis can result in an intra-abdominal abscess, peritonitis, and/or shock, the development of a pyogenic hepatic abscess is extremely rare.

**Case presentation:**

We present the case of a 2-year-old male who initially presented to the emergency department (ED) with fever and non-specific abdominal pain and was diagnosed with a urinary tract infection (UTI). He returned to the ED days later with rigors, worsening abdominal pain, and was diagnosed with a pyogenic hepatic abscess secondary to an ascending retrocecal appendicitis. In our patient, he did not just have a UTI with cultures growing *Escherichia coli*, but a hepatic abscess that was polymicrobial. He was started on broad-spectrum antibiotics and a 10 French pigtail catheter was placed. The patient was ultimately discharged on day 8 with continued antibiotics. After his antibiotic course, he underwent an elective laparoscopy appendectomy and is currently doing well post-operatively.

**Conclusion:**

Our case report illustrates the significance in identifying atypical features of appendicitis, broadening the differential of non-specific abdominal pain in pediatric patients, and depending on the clinical situation, ruling out other potential intra-abdominal infections even in the presence of a true urinary tract infection.

## Introduction

Appendicitis is common in children, occurring in 7% of the total population with a mean age between 8 and 10 years old [1]. In the case of the rare development of a potentially life-threatening pyogenic hepatic abscess, the clinical presentation is atypical and complex, with a reported annual incidence of < 0.03%, with < 10% of cases the result of appendicitis [2]. It is for this reason that an ascending retrocecal appendicitis may be clinically indistinguishable from acute pathology related to the liver, biliary tree, gallbladder, right kidney, and urinary tract.

## Case report

A 2-year-old male with no significant past medical history presented to the ED for cough, congestion, rhinorrhea, and intermittent fever for 3-day duration. The patient had no recent travel, was fully immunized, and was on no medications. His parents report multiple loose, brown, non-bloody episodes of diarrhea for the past 1 day with associated decreased oral intake. Other associated symptoms included non-specific bilateral flank pain, abdominal pain, and body aches. After multiple episodes of vomiting throughout the night and discovery of a temperature of 100.3 °F at home, the family brought the patient to the ED. Vitals on arrival were as follows: 98.2 °F (ibuprofen noted to be given prior to arrival), heart rate 90 beats/min, respiratory rate 25 breaths/min, and an oxygen saturation of 100% on room air. Physical exam revealed a well-appearing male in no acute distress. His nose exhibited clear rhinorrhea. Examination of the posterior pharynx revealed mild erythema with a clear post-nasal drip. His abdominal exam revealed diffuse non-specific tenderness, however, without distention or any surgical signs. Chest radiography revealed bilateral perihilar peribronchial cuffing. A point of care glucose was 194. Urinalysis revealed a specific gravity of 1.008, pH 6.5, ketones 40 mg/dL, 11–24 white blood cells/hpf, +nitrites, 2+ leukocyte esterase, and 2+ bacteria. Influenza and respiratory syncytial virus (RSV) detection by PCR was negative. He received intravenous fluids, was able to tolerate liquids/food in the ED, and was discharged home on trimethoprim/sulfamethoxazole (Septra) for a urinary tract infection.

Over the next few days, the family noted increasing abdominal pain and localized right flank pain with associated chills and nausea. Six days after his initial ED visit, the family reported he appeared to be shaking and had a decreased activity level, prompting a second visit to the ED. Vitals on arrival were as follows: 102.9 °F, heart rate 110 beats/min, respiratory rate 28 breaths/min, and an oxygen saturation of 100% on room air. On physical exam, his extremities felt cold without cyanosis, however, with a capillary refill of 3–5 s. He had voluntary guarding to the right upper and lower quadrants. His right flank exhibited costovertebral angle tenderness. His remaining physical exam including examination of his skin and genitourinary region was unremarkable. Code sepsis was activated based on vitals and physical exam. A total of 20 cc/kg fluid resuscitation was initiated, and a blood culture was drawn. Laboratory evaluation was notable for a white blood cell count (WBC) of 22.5 (5–15 bil/L), lactic acid of 2.3 (0.5–2.2 mmol/L), sodium of 131 (138–145 mmol/L), potassium 3.3 (3.5–5.2 mmol/L), chloride 92 (98–110 mmol/L), carbon dioxide 22 (20–29 mmol/L), glucose of 112 (60–99 mg/dL), C-reactive protein (CRP) of 23.9 (0.0–0.8 mg/dL), and an erythrocyte sedimentation rate (ESR) of 79 (0–15 mm/h). All remaining laboratory evaluation was unremarkable, including a hepatic function panel. Abdominal radiography was also negative for the acute process.

**Fig. 1:**
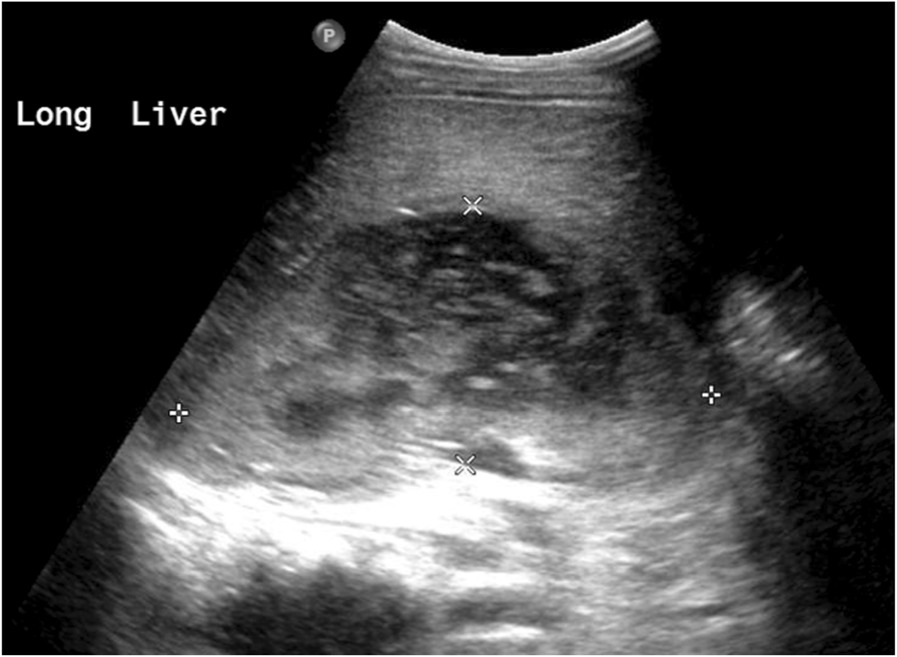
Ultrasound of the abdomen revealing a hypoechoic avascular area within the right upper quadrant. This complex fluid collection appears to have an intimate relationship with the liver parenchyma.

**Fig. 2:**
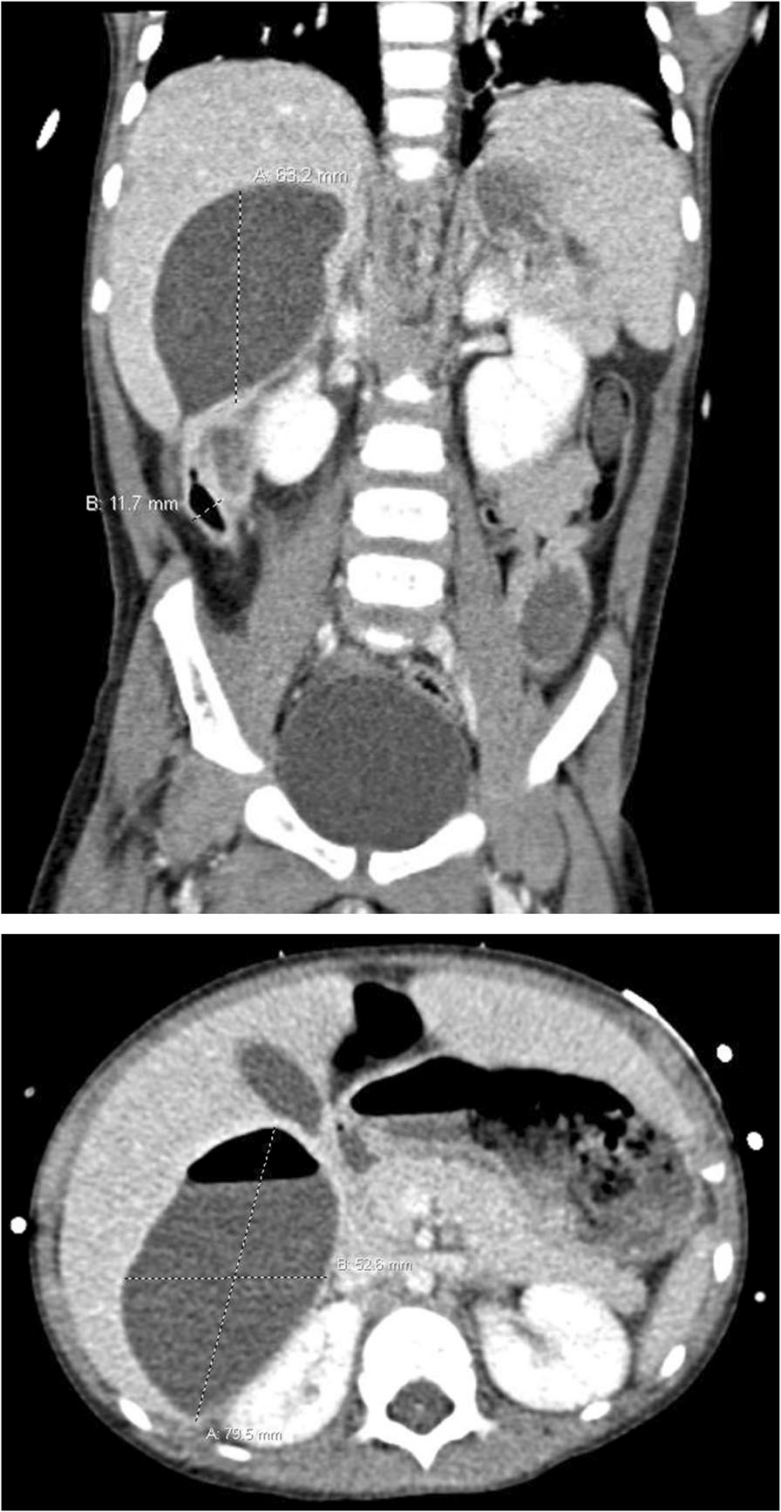
CT of the abdomen/pelvis with intravenous contrast revealing a retrocecal dilated appendix coursing cranially with adjacent inflammatory changes. Additionally, a large air-filled fluid collection is seen within the posterior right hepatic lobe measuring 9.0 × 5.3 × 6.3 cm. These findings are suspicious for an intrahepatic abscess

An ultrasound of the appendix was performed with unsuccessful visualization of the appendix. Given his positive urinalysis and clinical deterioration, suspicion arose for possible acute pyelonephritis, for which a complete ultrasound of the abdomen was done (Fig. 1), revealing a hypoechoic avascular area within the right upper quadrant. This complex fluid collection appeared to have an intimate relationship with the liver parenchyma. Subsequent CT of the abdomen/pelvis with intravenous contrast (Fig. 2) revealed a retrocecal dilated appendix coursing cranially with adjacent inflammatory changes. Additionally, a focus of air and fluid was seen within the posterior right hepatic lobe measuring 9.0 × 5.3 × 6.3 cm. These findings were suggestive of an intrahepatic abscess, possibly the result of acute appendicitis. The patient was started on intravenous piperacillin/tazobactam (100 mg/kg/dose q8h) for both a urinary tract infection and acute appendicitis with development of a hepatic abscess. He was transferred to a tertiary care facility with pediatric general surgery capabilities.

On day 2, the urine culture that was obtained by catheterization grew 10,000–50,000 CFU/mL of *Escherichia coli*. In addition, he underwent ultrasound-guided placement of a 10 French pigtail catheter into the intrahepatic abscess with resulting 100 mL of purulent drainage removed. By day 3, the patient was clinically improving with resolution of fever and nausea. Gram stain of the abscess fluid revealed Gram-positive cocci/Gram-positive bacilli consistent with a polymicrobial infection. Deep wound culture grew *Pseudomonas aeruginosa*. Anaerobic culture grew *Bacteroides fragilis* and *Peptostreptococcus* with susceptibilities to piperacillin/tazobactam. A PICC line was placed for outpatient antibiotic infusions. His percutaneous drain was removed on day 7 and repeat imaging demonstrated resolution of the intrahepatic abscess. He was discharged on day 8 with outpatient pediatric infectious disease and general surgery follow-up and continued antibiotic therapy for 4 weeks with weekly outpatient laboratory evaluation. After completion of his antibiotic course, the patient returned for an elective laparoscopic appendectomy. At his most recent post-operative visit, the patient is doing well.

## Discussion

Acute appendicitis is a common surgical condition that is associated with a low morbidity and mortality. Diagnosing appendicitis within the pediatric population can be challenging, rather it be a neonate presenting with irritability, a toddler presenting with flank pain, to a multitude of diverse clinical presentations, often mimicking a large differential. The classic presentation of appendicitis involves periumbilical pain with migration to the right lower quadrant and associated symptoms of nausea, vomiting, and fever. However, this presentation is only seen in 40% of pediatric patients < 12 years of age [3].

Since the appendix may occupy various positions in relation to the cecum, including the retrocecal region, patients with an ascending retrocecal appendicitis present to the ED with signs and symptoms that are often atypical and give rise to unusual presentations. Symptoms in these cases may mimic other disease processes including a viral syndrome, a urinary tract infection, acute cholecystitis, intussusception, pyelonephritis, ureterolithiasis/obstructive uropathy, and diverticulitis, to name a few [4]. This can result in a delayed diagnosis, increased incidence of complications, and increased morbidity and mortality. As such, a patient’s symptomatology will be determined based on the location and spread of inflammation with atypical presentations related to the location of the appendix. A large retrospective study was performed over an 8-year time frame involving 1670 patients with surgically proven appendicitis. Approximately 2% of those patients were diagnosed with an ascending retrocecal appendicitis with symptoms including right lower quadrant abdominal pain (49%), right flank pain (24%), right upper quadrant abdominal pain (18%), and periumbilical pain (15%) [5].

Cases of an ascending retrocecal appendicitis can result in the formation of an extremely rare and potentially life-threatening pyogenic hepatic abscess [4]. The abscess has a reported incidence of < 0.03%, with < 10% of cases secondary to appendicitis [2]. The development of this abscess has been attributed to the spread of bacteria or septic thrombi associated with the portal vein and its tributaries [6]. Acute appendicitis with ileocecal vein thrombophlebitis is considered a potential cause. Other causes include cholangitis, trauma to the liver, hematogenous spread from distal infections (i.e., osteomyelitis), and lymphatic spread from nearby infections (diverticulitis, ulcerative colitis, and suppurative pancreatitis) [6]. Common causative agents that have been associated with a pyogenic hepatic abscess include *E. coli*, *Staphylococcus aureus*, *Streptococcus* species, *Klebsiella pneumoniae*, *Pseudomonas aeruginosa*, *Entamoeba histolytica*, and *Bacteroides fragilis* [2, 7]. Furthermore, the signs and symptoms of a pyogenic hepatic abscess secondary to a retrocecal appendicitis range from non-specific abdominal pain, flank pain, leukocytosis, and fever, to abdominal distention, peritonitis, and shock [2]. It is for this reason that it may be clinically indistinguishable from acute pathology related to the liver, biliary tree, gallbladder, right kidney, urinary tract, and intestines [4].

While the morbidity and mortality of acute appendicitis is low, the mortality in the setting of a pyogenic hepatic abscess if left untreated is 100%. With early recognition and treatment (systemic antibiotics, percutaneous drain, and operative intervention), the mortality decreases to 15–20% [6, 8]. A thorough clinical history, laboratory evaluation, and appropriate imaging become imperative in recognition of not just a potential retrocecal appendicitis but also the additional presence of a pyogenic hepatic abscess. Two scoring systems that have been used to aid clinicians in diagnosing acute appendicitis are the Alvarado score and the pediatric appendicitis score. As published in the *Journal of Pediatric Emergency Care*, neither score has adequate predictive value and should not be utilized as an exclusive standard in diagnosing pediatric acute appendicitis. While the absence of leukocytosis, neutrophilia, and a normal CRP would suggest a lower likelihood of acute appendicitis, this does not rule out the diagnosis [9].

A prospective and retrospective study, as published in the *Journal of Pediatric Emergency Care*, involved 722 pediatric ED patients with non-traumatic abdominal pain. An elevated white blood cell count and a left shift were found to be independently and strongly associated with appendicitis in patients at least 1 year old. They found that a leukocytosis or presence of a left shift has a sensitivity of 79% for appendicitis, while the presence of both a leukocytosis and a left shift has a sensitivity of 94% [9]. Additionally, The Royal College of Surgeons of England published a prospective study involving the use of WBC and CRP in the diagnostic accuracy of acute appendicitis [1]. While the study included 98 patients total, it only included 20 pediatric patients. The authors concluded that a WBC < 11,000 bil/L and a normal CRP had a negative predictive value of 100% in patients < 18 years old [1]. However, the number of pediatric patients in this study was small and further publications since this one have had different results. A retrospective cohort study, published in the *Journal of the Royal Society of Medicine*, involved 297 pediatric and adult patients at 2 hospitals with histologically proven acute appendicitis. They were evaluated in terms of inflammatory markers. Overall, 17 patients (5.7%) had acute appendicitis with a normal WBC and CRP.

Additional testing includes obtaining a urinalysis, often done to rule out an alternative diagnosis. However, up to 25% of patients with appendicitis present with sterile pyuria on urinalysis [3]. Sterile pyuria involves the presence of leukocytes within the urine with the absence of bacteria. One study published in the Academic Surgical Congress by the University of Oklahoma College of Medicine involved a retrospective review of 219 pediatric surgical patients (ages 2–14) that underwent an appendectomy. Approximately 25% of those patients exhibited sterile pyuria. While it can be found in other disease processes like diverticulitis, sexually transmitted diseases, nephrolithiasis, and genitourinary tuberculosis, caution should be taken in pediatric patients that present with abdominal pain and found to have sterile pyuria [10]. Our patient did not present with sterile pyuria, rather a urinary tract infection with the urine culture growing *E. coli.* Our case presented clinicians with a unique challenge, demonstrating that suspicion for a potential additional intra-abdominal infection should still be present even in the presence of a urinary tract infection and pyuria.

Lastly, while ultrasound is often utilized in pediatric patients to assess for suspected appendicitis, it requires expertise and is often operator dependent. CT, however, is more sensitive and specific for appendicitis and is considered the gold standard. Furthermore, CT is useful in evaluating those patients with non-specific clinical findings, abnormal ultrasound, and atypical right upper quadrant abdominal pain/flank pain to rule out the possibility of a retrocecal appendicitis.

## Conclusion

Diagnosing acute appendicitis in pediatrics can be truly challenging, rather it be a neonate with irritability, a toddler with non-specific flank pain, to various atypical clinical presentations that mimic other disease processes. Depending on the location of the appendix, the classic presentation (right lower quadrant abdominal pain, nausea/vomiting, and fever) may not be present at all. In those patients that go on to develop the extremely rare complication and potentially life-threatening pyogenic hepatic abscess, the clinical presentation continues to diversify. Ultimately, this can result in a delayed diagnosis. As such, creating a broad differential of pediatric abdominal pain, maintaining a high index of clinical suspicion, thorough history/laboratory evaluation, and appropriate imaging are crucial in reducing morbidity and mortality.

## Data Availability

Data sharing is not applicable to this article as no datasets were generated or analyzed during the current study.
